# Evaluating the performance of convolutional neural networks with direct acyclic graph architectures in automatic segmentation of breast lesion in US images

**DOI:** 10.1186/s12880-019-0389-2

**Published:** 2019-11-08

**Authors:** Marly Guimarães Fernandes Costa, João Paulo Mendes Campos, Gustavo de Aquino e Aquino, Wagner Coelho de Albuquerque Pereira, Cícero Ferreira Fernandes Costa Filho

**Affiliations:** 1 Centro de Tecnologia Eletrônica e da Informação/Universidade Federal do Amazonas, Av. General Rodrigo Otávio Jordão Ramos, 3000, Aleixo, Campus Universitário – Setor Norte, Pavilhão Ceteli, Manaus, AM CEP: 69077-000 Brazil; 2 Programa de Engenharia Biomédica/COPPE/Universidade Federal do Rio de Janeiro, Rio de Janeiro, Brazil

**Keywords:** Breast lesion, Ultrasound images, Convolutional neural networks

## Abstract

**Background:**

Outlining lesion contours in Ultra Sound (US) breast images is an important step in breast cancer diagnosis. Malignant lesions infiltrate the surrounding tissue, generating irregular contours, with spiculation and angulated margins, whereas benign lesions produce contours with a smooth outline and elliptical shape. In breast imaging, the majority of the existing publications in the literature focus on using Convolutional Neural Networks (CNNs) for segmentation and classification of lesions in mammographic images. In this study our main objective is to assess the ability of CNNs in detecting contour irregularities in breast lesions in US images.

**Methods:**

In this study we compare the performance of two CNNs with Direct Acyclic Graph (DAG) architecture and one CNN with a series architecture for breast lesion segmentation in US images. DAG and series architectures are both feedforward networks. The difference is that a DAG architecture could have more than one path between the first layer and end layer, whereas a series architecture has only one path from the beginning layer to the end layer. The CNN architectures were evaluated with two datasets.

**Results:**

With the more complex DAG architecture, the following mean values were obtained for the metrics used to evaluate the segmented contours: global accuracy: 0.956; IOU: 0.876; F measure: 68.77%; Dice coefficient: 0.892.

**Conclusion:**

The CNN DAG architecture shows the best metric values used for quantitatively evaluating the segmented contours compared with the gold-standard contours. The segmented contours obtained with this architecture also have more details and irregularities, like the gold-standard contours.

## Background

Breast cancer is one of the leading causes of death among women under 40 years old [1]. According to the World Cancer Report, 2018, lung and female breast cancers are the leading types worldwide in terms of the number of new cases of cancers among women [2]. Studies have shown that detection of early-stage breast cancers, followed by appropriate treatment, was responsible for a 38% drop in the mortality rate from 1989 to 2014 [1]. Digital mammography (DM) and Ultrasound (US) are two commonly used techniques for breast lesion detection [3]. Although DM is considered the most effective technique [3], US imaging has the advantage of being safer, more versatile and sensitive to lesions located in dense areas, normally found in young women, and where lesions have an attenuation similar to the dense tissue. Therefore, they can be hidden by the surrounding tissue [4]. US imaging is heavily dependent on radiologist experience, compared to DM.

Outlining lesion contours in US breast images is an important step in breast cancer diagnosis. Malignant lesions infiltrate the surrounding tissue, generating irregular contours, with spiculation and angulated margins, while benign lesions produce contours with a smooth outline and elliptical shape [4]. On the other hand, low-contrast images associated with speckle noise generate spurious borders hampering lesion outline and hinder accurate diagnosis.

Spurred on by the success of machine learning and image processing in computer vision applications, many attempts have been made to build Computer-Aided Diagnosis (CAD) systems for breast lesion segmentation [5–9].

Daoud et al. [5] used support vector machines with texture input variables, for segmenting breast lesions in US images. The dataset consists of 50 breast US images with sizes of 418 × 566 pixels. The authors obtained the following results: True Positive Fraction = 91.13% ± 4.06%, False Positive Fraction = 8.87% ± 4.06% and False Negative Fraction = 15.58% ± 7.13%. In [6], a different approach was taken, using graph cuts and level set. However, the authors do not present quantitative results. In Jiang et al. [7], the authors used the algorithm of random walks to breast lesion segmentation in a dataset with 112 US images segmented by medical specialists. The authors obtained the following results: Accuracy = 87.5%, Sensitivity = 88.8% and Specificity = 84.4%. In [8], the dataset consists of only of 30 images and the authors used self-organized maps associated with finite impulse response filters for breast US images segmentation. The authors obtained the following results: True Positives (TP) = 93.24%, False Positives (FP) = 8.41% and Intersection over Union (IoU) = 86.95%. In [9], the authors used fuzzy histogram equalization for improving the US image contrast and the random forest classifier for breast lesion segmentation. The authors do not present quantitative results.

Considering the huge popularity of Deep Learning and, in particular, of CNNs in segmenting and classifying objects, the following question naturally arises: Can a CNN, using a relatively small dataset, such as those available in medical datasets, outperform traditional machine learning techniques in segmentation of breast tumors in US imaging?

According to Yap et al. [10], in breast imaging, a large number of recent publications concentrate on using CNNs for mammography. Dhungel et al. [11] addresses the problem of mass segmentation using deep learning; Mordang et al. [12] used CNNs in microcalcification detection. Recently, Ahn et al. [13] address the problem of breast density estimation using CNNs. Only the study of Yap et al. [10] focuses on CNNs for automatic segmentation of breast lesions in US images. The authors compared the performance of three CNN architectures, Le-Net [14], U-Net [15] and Fully Convolutional Network (FCN) Alex-Net [16] with three machine learning techniques, Rule-Based Region Ranking, Multifractal Filtering and Radial Gradient Index filtering. Two datasets, dataset A with 306 breast US images and dataset B with 163 breast US images were used in this comparison. According to the authors, considering the parameters false positives/image and F-measure, FCN-AlexNet obtained the best performance for dataset A and the Patch-based LeNet achieved the best performance for Dataset B. The authors conclude that the CNN architectures evaluated outperform traditional machine learning techniques in breast lesion segmentation in US imaging.

In this study our main objective is to assess the ability of CNNs in detecting contour irregularities in breast lesions in US images. With this aim, we propose two CNNs with DAG architectures and compare the performance of these proposed architectures with a proposed series architecture. DAG and series architectures are both feedforward networks. The difference is that a DAG architecture could have more than one path from the first layer and end layer, while a series architecture has only one path from first layer to end layer. When applied to image processing, DAG architectures aggregate information of pixel localization contained in initial layers into final layers. In semantic tasks, it is expected that this procedure enhances fine image details. Therefore, it is expected that DAG architectures will improve breast lesion segmentation in US images.

## Methods

### Work environment

Experiments were performed in Matlab 2017b (9.3.0.713579). The computer used was equipped with a DELL® motherboard with 128 GB RAM, a Intel® Core™ i5-7200U CPU @ 2.50GHz. The graphics processing unit used was a Nvidia GeForce 940MX, with 4GB RAM and 384 CUDA cores. The computer operating system was Windows 10.

### Input dataset

Two datasets were used in this work, dataset A and dataset B. Dataset A is composed of Breast Ultrasound Images (BUS) provided by Researchers from the Biomedical Engineering Graduate Program of Federal University of Rio de Janeiro – Brazil. The BUS images were acquired during routine breast diagnosis procedures, by several radiologists, at the Cancer National Institute (Rio de Janeiro, Brazil) from different patients using different old ultrasound equipment. The patient information, contained in the images, was excluded by an image cropping step, resulting in different image sizes. For each image, one experienced radiologist manually delineated all lesions. According to histopathological analysis there are 179 malignant lesions and 208 benign lesions. Each image is from a different patient.

Dataset B was collected in 2012 from the UDIAT Diagnostic Centre of the Parc Taulí Corporation, Sabadell (Spain) with a Siemens ACUSON Sequoia C512 system 17 L5 HD linear array transducer (8.5 MHz). The dataset consists of 163 images from different women with different image sizes. Within the 163 lesion images, 53 were images with cancerous masses and 110 with benign lesions [10].

Due to hardware limitations, all images of both databases were resized to 160 × 160 pixels or to 320 × 320 pixels. We evaluated the following sets: Dataset A: cropped image resized to 160 × 160; Dataset B: cropped image resized to 160 × 160, original image resized to 160 × 160, original image resized to 320 × 320.

**Fig. 1:**
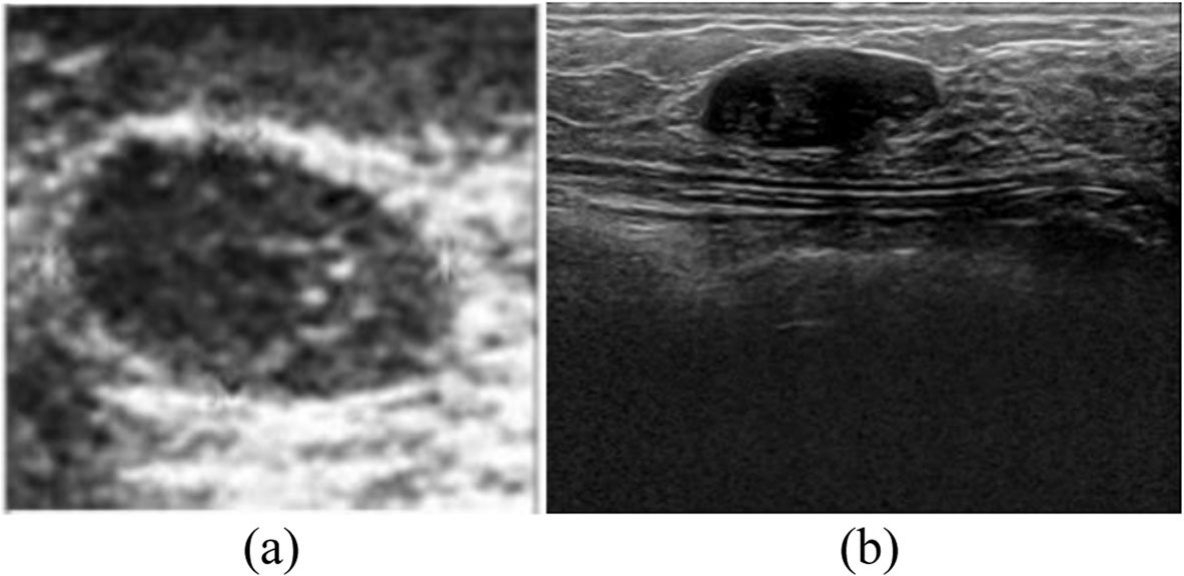
**a** Example of a cropped image from Dataset A and **b** Example of original image from Dataset B

Figure 1a shows an original image from dataset A, while Fig. 1b shows an original image from dataset B. As can be seen in Fig. 1, the image from dataset A is noisy. Due to cropping, the lesion looks larger. On the other hand, the image of dataset B is high quality.

A subset of Dataset A, composed of 200 images, was also used by Infantosi et al. [16] for lesion segmentation. The authors made the lesion segmentation with morphologic operators and a Gaussian Function Constraint.

A subset of Dataset A, composed of 50 images, was also used by Gomez et al. [17]. The authors made the lesion segmentation using a marker-controlled watershed transformation.

Dataset B was used for lesion detection (not lesion segmentation) by Yap et al. [10].

### Semantic segmentation

Semantic segmentation is formulated as a discrete labelling problem that assigns each pixel *x*_*i*_ of an image to a label *l*_*i*_ from a fixed set *ϕ*. Given a set of pixels *X* = {*x*_1_, *x*_2_…*x*_*n*_} the task is to predict the set of labels *L* = {*l*_1_, *l*_2_…*l*_*n*_}, taking values from *ϕ*. In the segmentation problem solved in this work, the set *ϕ* is comprised of two values, *ϕ* = {0, 1}. The label 0 must be assigned to pixels that belong to the background and the label 1 must be assigned to pixels that belong to a lesion. The Convolutional Neural Networks used in this work make a semantic segmentation. Binary images are generated in their output with the same size as those presented in the input and with pixels labeled with values belonging to the *ϕ* set.

### Convolutional neural network architectures

**Fig. 2:**
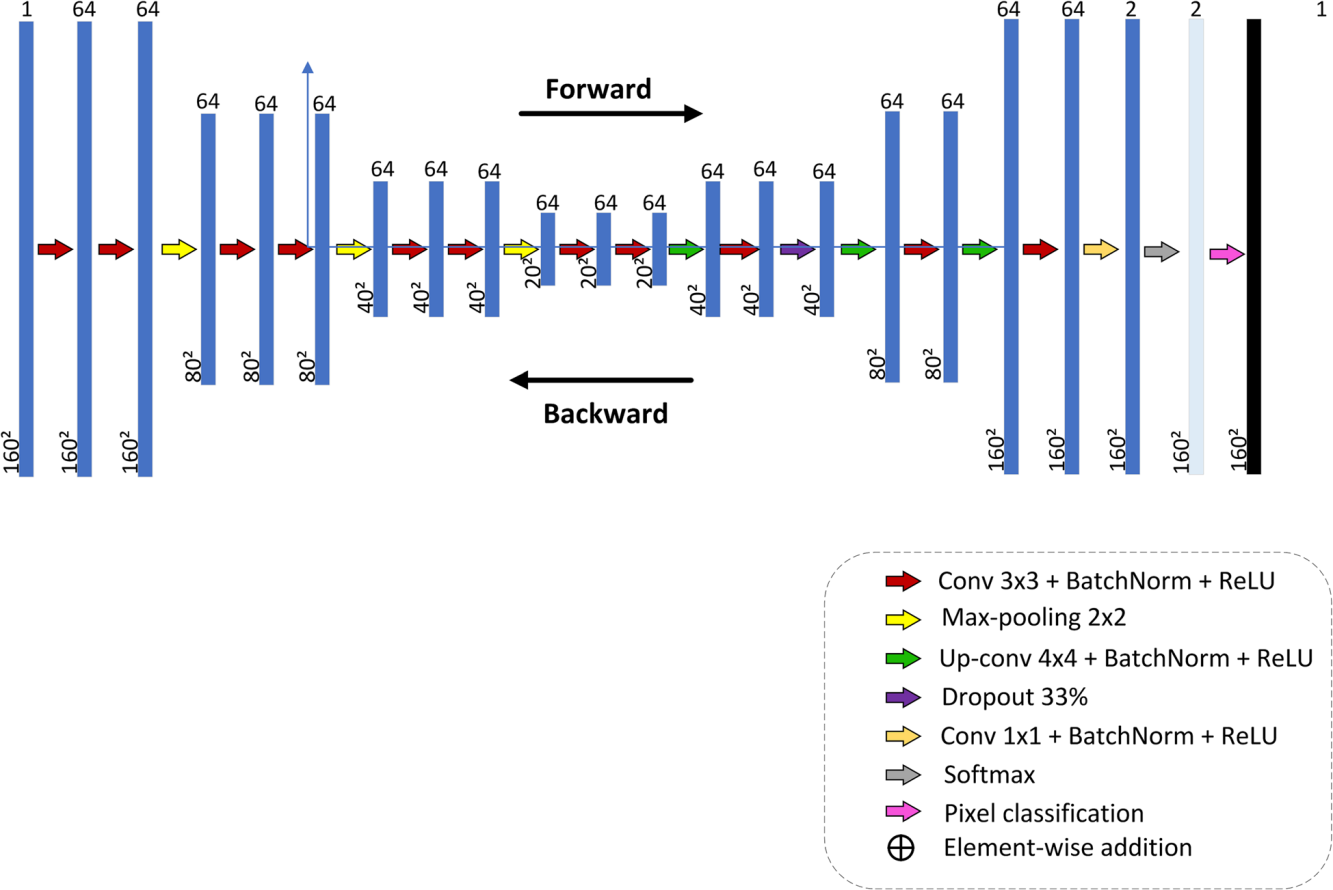
Series CNN architecture proposed for CNN segmentation

The first CNN architecture proposed in this study for breast lesion semantic segmentation in US image, CNN1, is a series architecture. In this architecture, the input of each layer is the output of the previous layer. A series architecture is always used in the studies of Roth et al. [18], the Pure CNN architecture, used for pancreas segmentation in CT images, and Shelhamer et al. [19], the FCN architecture, used for semantic segmentation in general. Figure 2 shows the proposed CNN1 architecture, obtained empirically through several experiments. We tried smaller architectures, but noticed the presence of some noise in the final image. The proposed architecture minimized the presence of noise in the final image. The following layer names are used to describe the network architecture: Convolutional (Conv), Batch Normalization (BatchNorm), Rectifier Linear Unit (ReLU), Maximum Pooling (MaxPooling), Deconvolution (Deconv). The overall net is formed by: (Conv64-BatchNorm-ReLU (2x) – MaxPooling) (3x) – Conv64-BatchNorm-ReLU(2x)–Deconv64-BatchNorm-ReLU-Conv64-BatchNorm-ReLU-Dropout-(Deconv64-BatchNorm-ReLU-Conv64-BatchNorm-ReLU) (2x)- MaxPooling- Conv2-BatchNorm-ReLU-Softmax–PixelClassification. Layers play two important roles with respect to the network operation as a whole: a forward pass that takes the inputs and calculates the outputs, and a backward pass that computes the gradients and adjusts the layer parameters in accordance with them. In the adopted deep learning framework, data input is regarded as a layer. In our case, the data type of input layer was the Matlab Image Datastore object, which manages a collection of image files, where each individual image fits in the memory, but the entire collection of images does not necessarily fit. The network contains eleven convolutional layers. The receptive fields of the first ten equal to 3 × 3 pixels and of the last one is 1 × 1 pixel; both padding and stride hyper-parameters are equal to one. All convolutional layers, except the last one, have 64 feature maps. The last convolutional layer produces two feature maps, since pixels will be classified in two classes: (0) background, (1) lesion. All weights of the convolution layers were initialized according to a Gaussian distribution with mean 0 and standard deviation of 0.01. The biases were initialized as constants, with zero as default value. During training, the weights of these convolutional layers are adjusted to identify visual features, such as edges, orientations or certain patterns in the images. Each convolutional layer is followed by a ReLU layer, which applies an activation function to neurons defined as f(x) = max (0, x), where x is a single neuron input. According to Krizhevsky et al. [20], the ReLU units accelerate network convergence. MaxPooling layers progressively reduce the input spatial size to reduce the number of parameters and computation in the network. All these MaxPooling layers have 2 × 2-sized filters applied with a stride of 2 and padding of 0, down sampling every depth slice in the input by 2 along both width and height, discarding 75% of the activations. The MAX operations take the maximum value from a 2 × 2-pixel region. The depth dimension remains unchanged. The Dropout layer reduces overfitting by preventing complex co-adaptations in training data. A Dropout ratio parameter sets the probability that any given unit is dropped. In this work, the dropout ratio parameter was set to 0.5. To speed up training of convolutional neural networks and reduce the sensitivity to network initialization, a Batch Normalization layer is used between convolutional layers and nonlinearities, such as ReLU layers. It normalizes each input channel across a mini-batch. The Deconvolution layers perform an up-sampling to obtain a predictive map of pixel classification with the same size as the input; in other words, it predicts the class to which each pixel belongs. All the Deconvolution layers use filters with receptive fields of 4 × 4 pixels. It works inversely to the convolutional layer. It reuses the convolution layer parameters, but in the opposite direction, that is, the padding is removed from the output rather than added to the input, and the stride results in an up-sampling rather than a sub-sampling.

The Softmax layer calculates both the softmax and the multinomial logistic loss operations, which saves time and improves numerical stability. It takes two inputs, the first one being the prediction of the prior layer (Conv2) and the second one being the label layer. It computes the loss function value, which is used by a backpropagation algorithm to calculate the gradients with respect to all weights in the network.

**Fig. 3:**
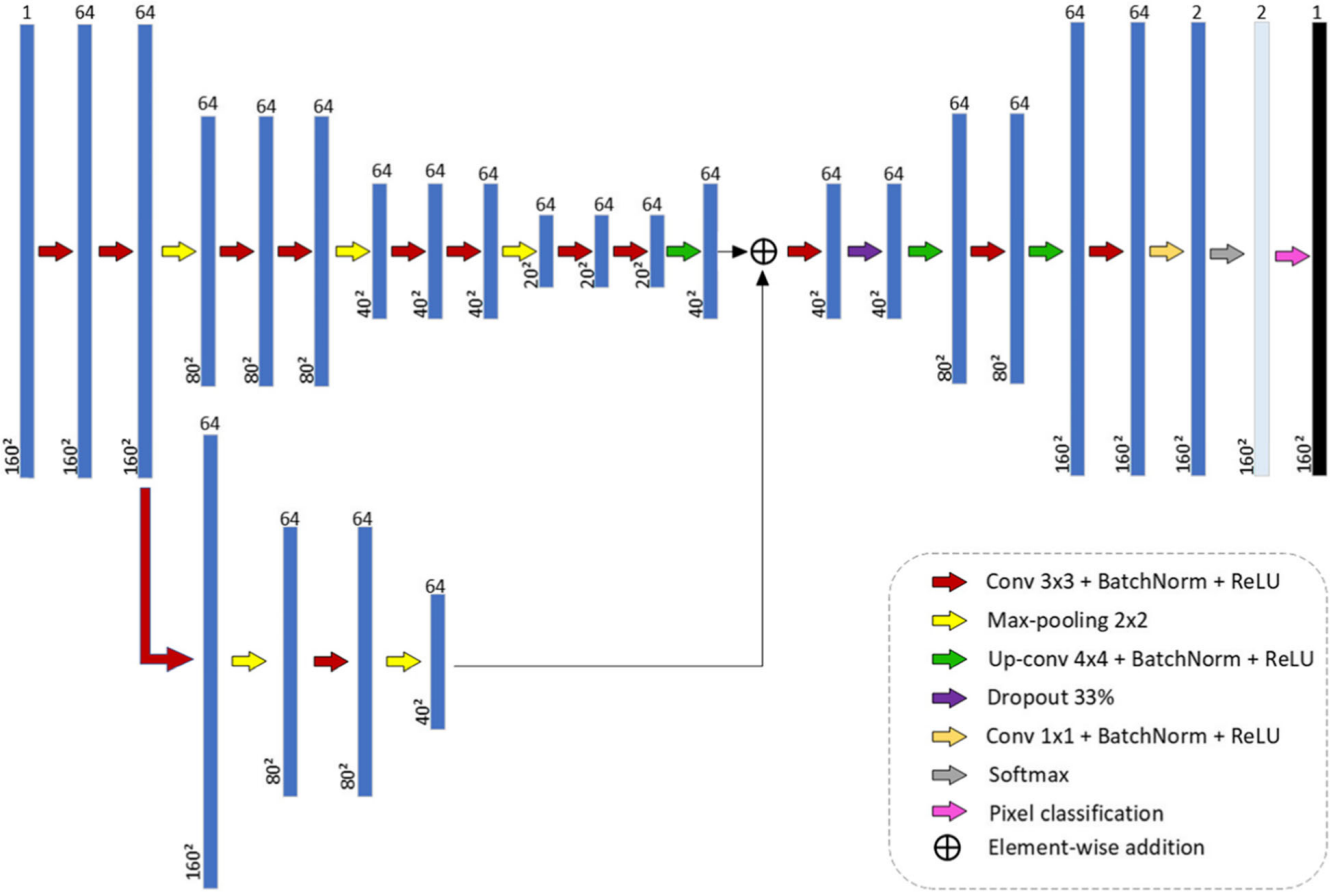
CNN2 architecture: first DAG architecture proposed for breast lesion segmentation in US image

**Fig. 4:**
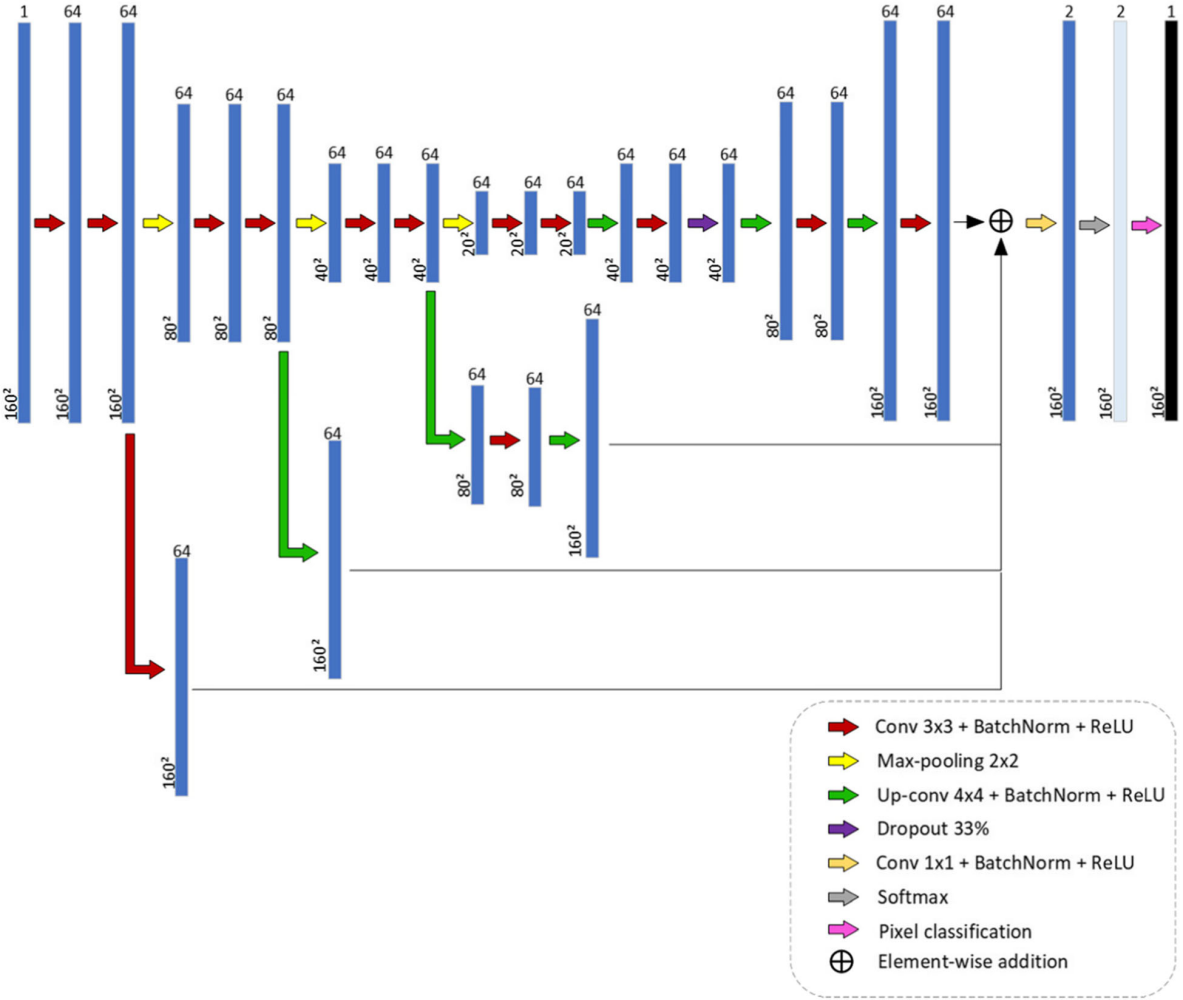
CNN3 architecture: second DAG architecture proposed for breast lesion segmentation in US images

The second and third CNN architectures proposed in this study for breast lesion segmentation in US image, CNN2 and CNN3, are DAG architectures. A DAG architecture has layers arranged as a directed acyclic graph. A DAG architecture is more complex than a series architecture, in which layers have inputs from multiple layers and outputs to multiple layers. When applied to image processing, these architectures aggregate information of pixel localization contained in initial layers into final layers. In semantic tasks, it is expected that this procedure enhances fine image details. The study of Chen et al. [21] used a DAG architecture to segment neuronal structures in Electron Microscope Images. Figures 3 and 4 show the two proposed DAG architectures. In both, the main network path is like CNN1.

In CNN2, information from the first sub-sampling module, after the second convolution operation, is aggregated before the convolution operation of the first up-sampling module. To implement aggregation, it is necessary that the data representation in both inputs be of the same size. Since the data dimension after the two convolution operations of the first sub-sampling module is 160 × 160, and after the deconvolution of the first up-sampling module is 40 × 40, a dimension reduction of the first one is necessary. This is accomplished with two MaxPooling operations with 2 × 2-sized filters.

In CNN3, information from the three sub-sampling modules, after the second convolution layer of each one, is aggregated before the last convolution layer, Conv2. The number of layers of CNN1, CNN2 and CNN3 is 52, 61 and 68, respectively.

We evaluated the performance of the proposed architectures applying various hyper parameter adjustments. For CNN training, the algorithm Stochastic Gradient Descent with Momentum (SGDM) was used. The SGDM algorithm might oscillate along the path of the steepest descent towards the optimum. Adding a momentum term to the parameter update is one way to reduce this oscillation [22]. The stochastic gradient descent with momentum update is.

1 $$ {\theta}_{l+1}={\theta}_l-\nabla E\left({\theta}_l\right)+\gamma \left({\theta}_l-{\theta}_{l-1}\right) $$

The momentum γ determines the contribution of the previous gradient step to the current iteration. *θ* is the learning rate and ∇*E* its gradient. The initial learning rate was set in 0.001 and the momentum in 0.9. Due to limitations in the Graphical Processor Unit memory, batch size was set to 5. Stop condition was set in 150 epochs. This maximum was selected observing the convergence of the CNN training.

A quantitative analysis of the performance of the three architectures is made using the following metrics: accuracy, global accuracy, IoU, Boundary F1 (BF) score and Dice Similarity Coefficient. The accuracy refers to proportion of pixels corrected classified per class, lesion or background, while the global accuracy refers to proportion of pixels corrected classified, regardless their class, lesion or background.

### Training, validation and testing

Both databases were divided into three subsets: training, validation and testing. In each database, 60% of the data was used for training, 20% for validation, and 20% for testing. For dataset A, this corresponds to 233, 77, and 77 images. For dataset B, this corresponds to 97, 33, and 33 images. The proportion of malignant and benign lesions in each subset reflected this same proportion. The validation step was used for selecting the CNN architecture with best performance. After choosing the architecture with the best performance in the validation set, the training and test set were merged, and a 5 cross-validation strategy was applied to evaluate it.

### Evaluation metrics

The following evaluation metrics were used: global accuracy, IoU, Dice coefficient and BF score. In the description of these evaluation metrics, we will use the following definitions: False Positives: pixels that belong to the background that were misclassified as belonging to lesions; False Negatives (FN): pixels that belong to lesions that were misclassified as belonging to the background; True Positive: pixels that belong to lesions that were correctly classified as belonging to lesions; True Negative (TN): pixels that belong to the background that were correctly classified as belonging to the background.

The global accuracy is the ratio between the pixels correctly classified, regardless of class, and the total number of pixels and is given in Eq. (2):

2 $$ global\ accuracy=\frac{TP+ TN}{TP+ TN+ FP+ FN} $$

The accuracy gives the proportion of corrected classified pixels in each class and is given in Eq. (3):

3 $$ accuracy=\frac{\left( TP/ TP+ FN\right)+\left( TN/ TN+ FP\right)}{2} $$

The IoU is a metric that penalizes the incorrect classification of pixels as lesions (FP) or as background (FN), and is given in Eq. (4):

4 $$ IoU=\frac{Lesion+ Background}{2} $$

Where:

5 $$ Lesion=\frac{TP}{TP+ FN+ FP} $$

6 $$ Background=\frac{TN}{TN+ FN+ FP} $$

The Weighted IoU is used when there is a disproportionate relation between the class sizes in the images, minimizing the penalty of wrong classifications in smaller classes. It is given in Eq. (7).

7 $$ Weighted\  IoU= Lesion\ weight\ x\  lesion+ Background\ weight\ x\  background $$

Where:

8 $$ Lesion\ weight=\frac{number\ of\ pixels\ belonging\ to\ lesion}{total\ number\ of\ pixels} $$

9 $$ Brakground\ weight=\frac{number\ of\ pixels\ belonging\ to\ lesion}{total\ number\ of\ pixels} $$

The Dice coefficient measures the proportion of pixels correctly classified as lesion, penalizing the incorrect classification (FP or FN), and is given in Eq. (10).

10 $$ Dice=\frac{2 TP}{2 TP+ FN+ FP} $$

The BF Score measures the alignment between the predicted borders and the gold standard one. It is given by a weighted harmonic mean of precision and recall, as shown in Eq. (11):

11 $$ BF\  score=\frac{2x\left( precision+ recall\right)}{precision+ recall} $$

## Results

**Fig. 5:**
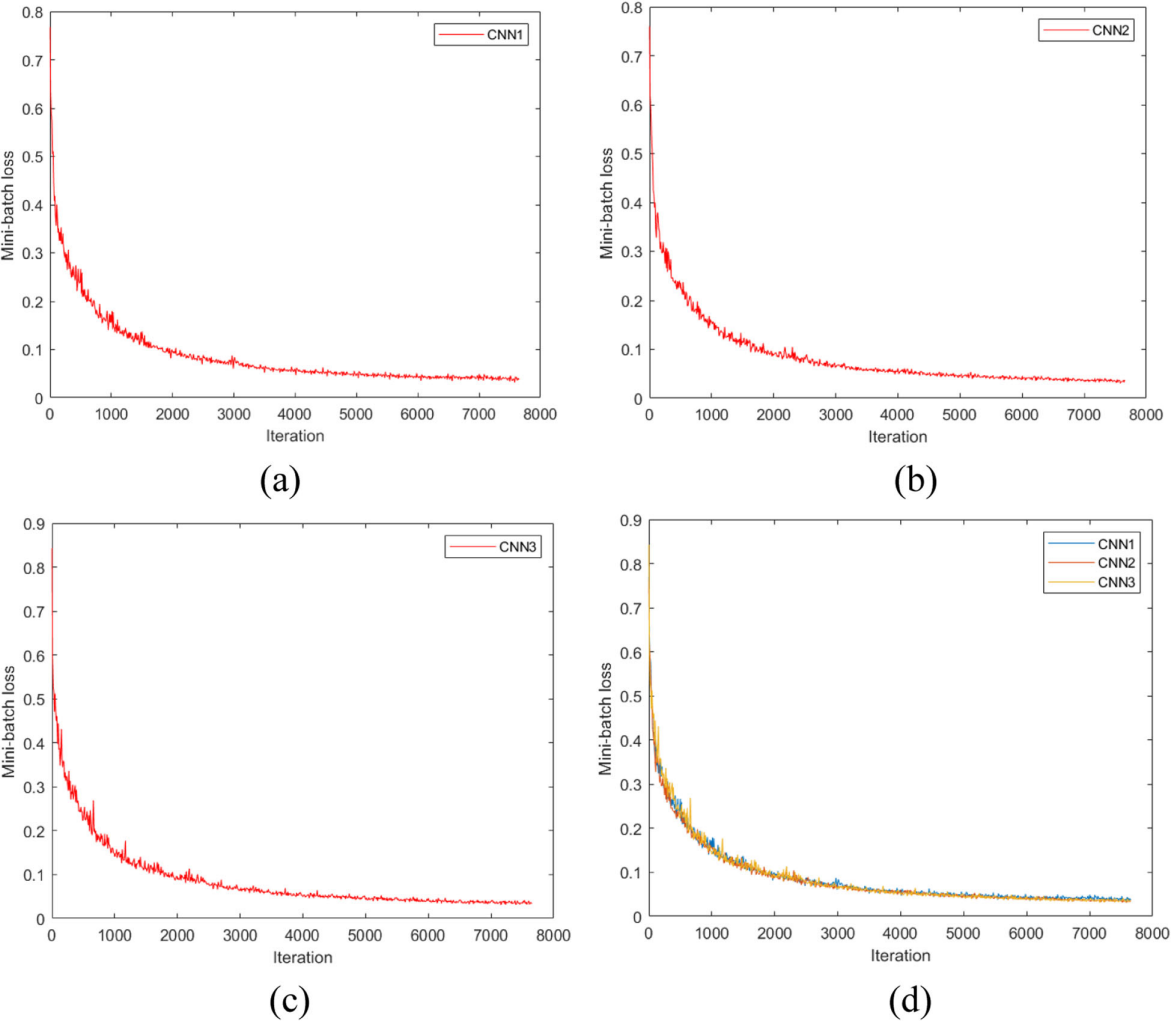
Mini-batch loss versus iteration: **a** CNN1; **b** CNN2; **c** CNN3; **d** comparison between CNN1, CNN2 and CNN3

Figure 5 shows the graphs of network convergence, using dataset A, with the SGDM optimization algorithm. In the *x* and *y* axes are the iterations and mini-batch loss values, respectively. During the training, the network weights are adjusted in order to decrease the mini-batch loss value, forcing the algorithm convergence to the minimum. As this is a stochastic process and the weight are randomly initialized, successive trainings on the same dataset do not result in equal weights at the end. As shown in Fig. 5d, the speed convergences of CNN1, CNN2 and CNN3 are almost the same. In these networks, a plateau is reached after 7000 iterations. With dataset A, the training times of CNN1, CNN2, CNN3 were 95′14″, 112′4″, 183′ 35″, respectively. These training times maintain a strong relationship with the CNN architecture sizes.

**Fig. 6:**
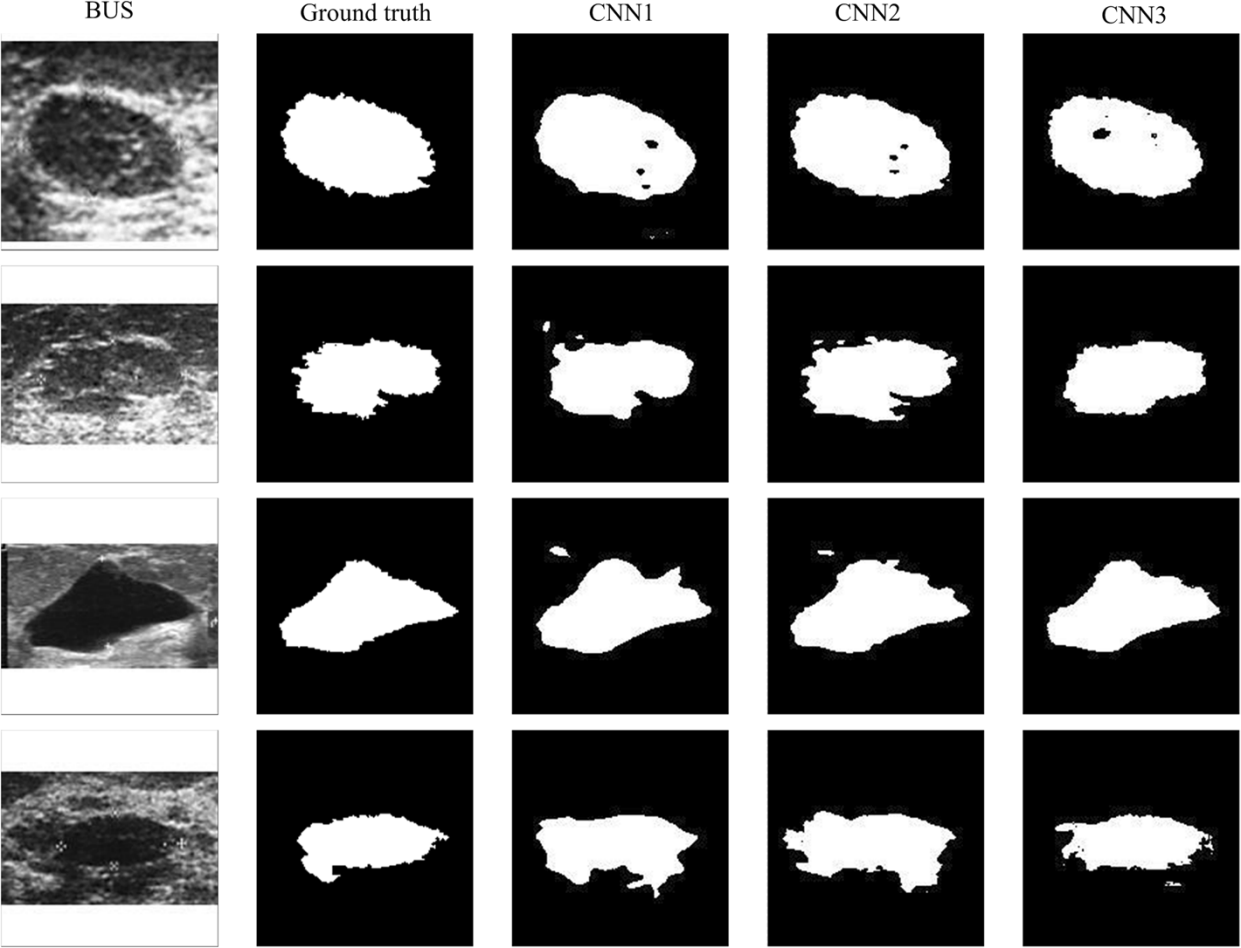
Examples of contours obtained by the three CNN architectures

Aiming at a qualitative analysis, Fig. 6 shows examples of segmentations performed by the three architectures in 3 breast lesions of dataset A. As observed, the contours obtained by CNN1 are smoother than the contours obtained by the DAG architectures. The architecture CNN1 sub-samples the input image with dimension of 160 × 160 pixels to 20 × 20 pixels and then up-samples to 160 × 160 in the final layers. In this process details of the lesion contours are lost, generating smooth contours such as those shown in Fig. 6. The contours obtained with the DAG architectures, CNN2 and CNN3 have more details and irregularities, like the gold-standard contours. The reason is that these architectures aggregate to the last layers information from initial layers, thus preserving pixel localization in the original image.

**Fig. 7:**
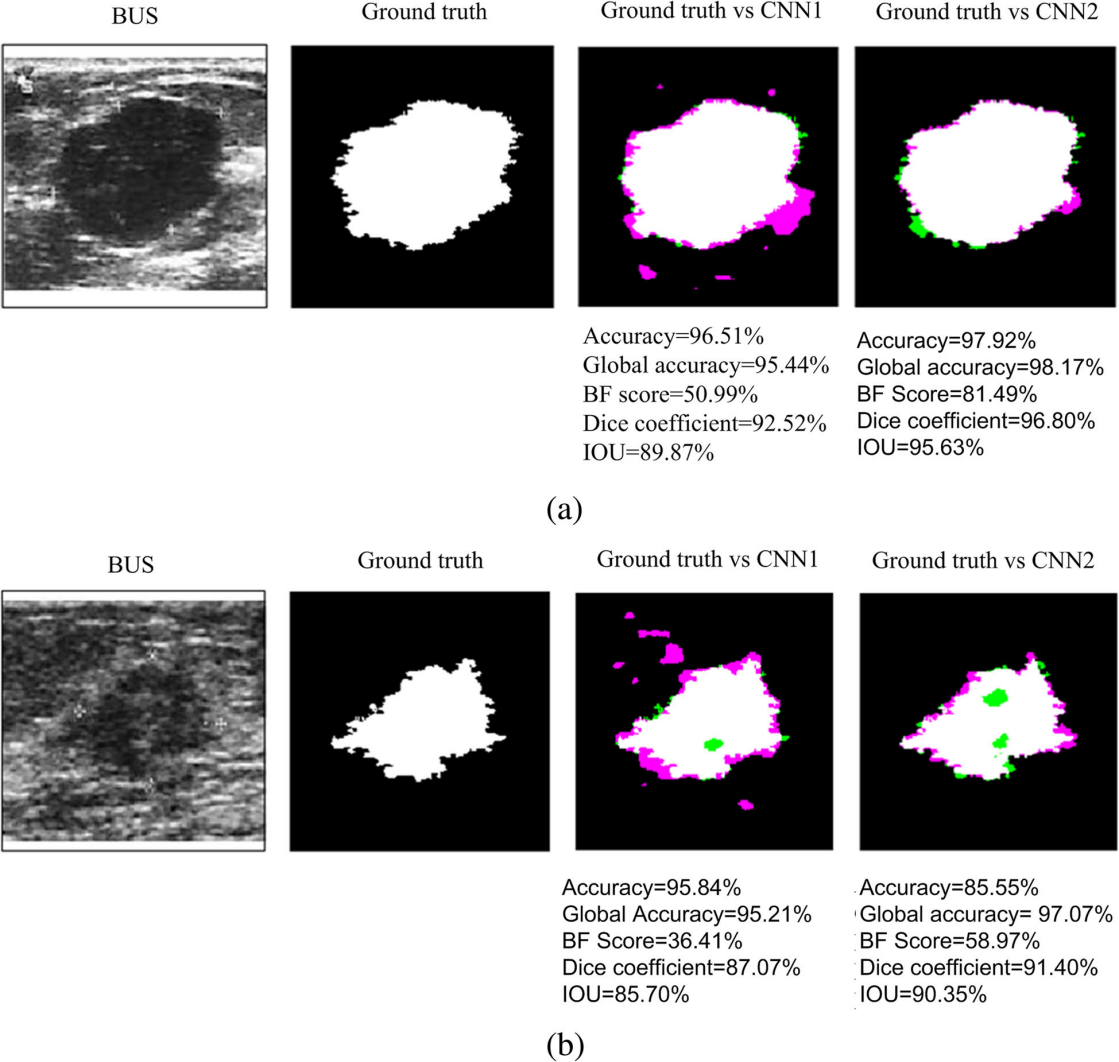
Quantitative analysis of two segmentations obtained with the three CNN architectures. **a** benign lesion; **b** malignant lesion. Pink color represents false positive pixels, while green color represents false negative pixels. Metric values for each contour are shown below each image

Figure 7a and b show a quantitative analysis of a benign and a malignant lesion, respectively, obtained from dataset A. The pink color shows false positive pixels (pixels outside the ground truth contour considered as inside, in the obtained contour), while green color shows false negative pixels. The contour with lower number of false positives and false negatives pixels is the one obtained with CNN3. Below each image the metrics of each contour are shown. As can be seen, the best metrics values are also obtained with CNN3.

Tables 1 and 2 show the results of the validation step, for datasets A and B. The best values are shown in boldface.

**Table 1:** Mean values of the metrics for the dataset A, using the validation set and cropped images resized to 160 × 160 pixels

CNN	Global Accuracy	Accuracy	IoU	Weighted IoU	BF Score	Dice Coefficient
CNN1						
Mean	0.904	0.916	0.766	0.843	0.472	0.776
Standard Deviation	0.045	0.047	0.095	0.063	0.085	0.119
CNN2						
Mean	0.895	0.917	0.759	0.835	0.479	0.770
Standard Deviation	0.063	0.046	0.117	0.081	0.112	0.141
CNN3						
Mean	0.935	0.919	0.819	0.886	0.553	0.829
Standard Deviation	0.030	0.050	0.076	0.050	0.103	0.009

**Table 2:** Mean values of the metrics for the dataset B, using the validation set and cropped images resized to 160 × 160 pixels

CNN	Global Accuracy	Accuracy	IoU	Weighted IoU	BF Score	Dice Coefficient
CNN1						
Mean	0.917	0.904	0.836	0.850	0.510	0.920
Standard Deviation	0.048	0.068	0.090	0.079	0.083	0.032
CNN2						
Mean	0.911	0.895	0.823	0.838	0.515	0.915
Standard Deviation	0.047	0.074	0.095	0.080	0.092	0.034
CNN3						
Mean	0.921	0.914	0.845	0.857	0.516	0.918
Standard Deviation	0.035	0.046	0.067	0.059	0.086	0.031

Comparing Tables 1 and 2, we notice that CNN3 presents the best values for all metrics and for both databases. The best values of global accuracy, mean accuracy, weighted IoU and mean BF score were obtained with dataset A, while the best values for mean IoU and Dice coefficient were obtained with dataset B.

The differences between the global accuracies in each dataset were evaluated using *t-student* hypothesis test. The calculated value of *t-student* test is compared with a critical value *t*_*c*_. The null hypothesis is rejected if or *t* ≥ *t*_*c*_ *or t* ≤  − *t*_*c*_. In the first case, the mean value is considered significantly higher, and, in the second case, significantly lower. In this study, a confidence level of 95%, 152 degrees of freedom were used for dataset A, corresponding to a critical value of *t*_*c*_ = 1.982. For dataset B we have 64 degrees of freedom, corresponding to a critical value of *t*_*c*_ = 2.000.

Comparing the results of CNN3 with CNN2 in dataset A, we obtained a *t-value* =5.062. This value is statistically significant. Comparing the results of CNN2 with CNN1 in dataset A, we obtained a *t-value* = − 1.026, not statistically significant. Comparing the results of CNN3 with CNN1 in dataset A, we obtained a *t-value* =5.062. This result is statistically significant. Comparing the results of CNN3 with CNN2 in dataset B, we obtained a *t-value* =1.078. This result is not statistically significant. Comparing the results of CNN2 with CNN1 in dataset A we obtained a *t-value* = − 0.605. This result is not statistically significant. Comparing the results of CNN3 with CNN1 in dataset A, we obtained a *t-value* =0.381. This result is not statistically significant.

Therefore, although the best metrics values are obtained with CNN3, the differences in Global Accuracies obtained with this network and with CNN2 and CNN1 are statistically significant only for dataset A.

Tables 3 and 4 shows results using CNN3 and cross-validation with 5 folders, for datasets A and B, respectively. The networks were trained and tested with cropped images resized to 160 × 160 pixels. Comparing Tables 3 and 4, we notice that the best values of all metrics were obtained with dataset A. The differences between the global accuracies were evaluated using *t-student* hypothesis test. The calculated value of *t-student* test is compared with a critical value *t*_*c*_. In this study, a confidence level of 99% and 5 degrees of freedom were used (5 folders), corresponding to a critical value of *t*_*c*_ = 4.032. We obtained a *t-value* =4.183. This value is statistically significant.

**Table 3:** Metrics values for cross-validation with 5 folders for dataset A, using cropped images resized for 160 × 160 pixels

Folder	Global Accuracy	Accuracy	IoU	Weighted IoU	BF Score	Dice Coefficient
1	0.952	0.944	0.864	0.913	0.679	0.877
2	0.969	0.961	0.904	0.942	0.754	0.916
3	0.936	0.944	0.834	0.887	0.603	0.850
4	0.961	0.947	0.884	0.923	0.704	0.904
5	0.964	0.954	0.894	0.932	0.724	0.915
Mean	0.956	0.950	0.876	0.920	0.693	0.918
Standard Deviation	0.011	0.006	0.025	0.019	0.051	0.025

**Table 4:** Metrics values for cross-validation with 5 folders for dataset B, using cropped images resized for 160 × 160 pixels

Folder	Global Accuracy	Accuracy	IoU	Weighted IoU	BF Score	Dice Coefficient
1	0.917	0.916	0.846	0.846	0.550	0.910
2	0.930	0.930	0.865	0.867	0.556	0.914
3	0.889	0.890	0.800	0.800	0.496	0.892
4	0.924	0.926	0.858	0.859	0.541	0.917
5	0.926	0.925	0.860	0.863	0.542	0.946
Mean	0.917	0.917	0.846	0.847	0.537	0.916
Standard Deviation	0.016	0.016	0.027	0.027	0.024	0.019

Tables 5 and 6 shows the results obtained using CNN3 and cross validation with 5 folders, for dataset B, with original images resized to 160 × 160 pixels and 320 × 320 pixels.

**Table 5:** Metrics values for cross-validation with 5 folders, for dataset B, using original images resized for 160 × 160 pixels

Folder	Global Accuracy	Accuracy	IoU	Weighted IoU	BF Score	Dice Coefficient
1	0.982	0.915	0.806	0.969	0.669	0.692
2	0.987	0.900	0.844	0.976	0.730	0.758
3	0.977	0.833	0.744	0.960	0.597	0.574
4	0.970	0.820	0.738	0.947	0.630	0.608
5	0.983	0.933	0.837	0.969	0.693	0.714
Mean	0.979	0.880	0.794	0.964	0.664	0.669
Standard Deviation	0.007	0.051	0.050	0.011	0.052	0.076

**Table 6:** Metrics values for cross-validation with 5 folders for dataset B, using original images resized for 320 × 320 pixels

Folder	Global Accuracy	Accuracy	IoU	Weighted IoU	BF Score	Dice Coefficient
1	0.918	0.903	0.811	0.853	0.501	0.820
2	0.887	0.875	0.756	0.806	0.443	0.799
3	0.921	0.909	0.811	0.811	0.490	0.838
4	0.900	0.867	0.772	0.773	0.476	0.789
5	0.918	0.911	0.805	0.773	0.507	0.871
Mean	0.909	0.893	0.791	0.803	0.483	0.823
Standard Deviation	0.014	0.020	0.025	0.033	0.025	0.032

Comparing Tables 4 and 5, we notice that the best values of mean accuracy, mean IoU and Dice coefficient were obtained with dataset B with cropped images resized to 160 × 160 pixels, while the best values for global accuracy, weighted IoU and mean BF score were obtained with dataset B, with original images resized to 160 × 160 pixels. The differences between the global accuracies were evaluated using *t-student* hypothesis test. The calculated value of *t-student* test is compared with a critical value *t*_*c*_. In this study, a confidence level of 95% and 8 degrees of freedom were used, corresponding to a critical value of *t*_*c*_ = 3.355. We obtained a *t-value* = − 7.938. This value is statistically significant.

Comparing Tables 5 and 6, we notice that the best values of global accuracy, mean IoU, weighted IoU and mean BF score were obtained with dataset B, with original images resized to 160 × 160 pixels, whereas the best values for Mean Accuracy and Dice Coefficient were obtained with dataset B, with original images resized to 320 × 320 pixels. The differences between the global accuracies were evaluated using *t-student* hypothesis test. The calculated value of *t-student* test is compared with a critical value *t*_*c*_. In this study, a confidence level of 95% and 8 degrees of freedom were used, corresponding to a critical value of *t*_*c*_ = 3.355. We obtained a *t-value* =10.000. This value is statistically significant.

## Discussion

The main advantage of CNNs compared with traditional machine learning techniques in segmentation and classification tasks, is that the former are fully automated, requiring no pre-processing for characteristic extraction.

The performance of CNNs is strongly dependent on the existence of large databases. This is a challenge for medical applications, since we have relatively small datasets in this research area. In previous studies published in the literature for breast lesion segmentation, it was shown (digital mammography [11–13] and US [10]) that, even with small datasets, CNN outperforms the traditional machine learning techniques in breast lesion segmentation and classification. Dataset A used in this study comprises 387 US images: 208 are benign lesions and 179 are malignant lesions. Compared with other datasets previously cited and used for breast lesion segmentation, 50 images [5], 112 images [6] and 30 images [8], dataset A is the larger one.

In deep learning, there is a plenty of CNN architectures that have been proposed for image segmentation and classification. It is impossible to evaluate all these architectures in each application. From previous knowledge of the characteristics of each architecture, it is possible to select an appropriate one, with tailored characteristics to solve a given problem.

In this study, the main task was to evaluate if CNN architectures could outline irregular contours, with spiculation and angulated margins, such as those found in US breast lesions images. Our choice was to use DAG architectures. From a previous knowledge of the performance of the DAG architecture in image segmentation applications, we knew that these architectures aggregate information of pixel localization contained in initial layers into final layers, preserving fine image details. In this study, the performance of the DAG architectures, compared with a series architecture, is superior, both qualitatively as quantitatively.

The comparison of the performance of CNN3, CNN2 and CNN1 in Tables 1 and 2 shows that CNN3 present best performance for all metrics in both datasets. However, the differences between global accuracies are only statistically significant in dataset A.

The comparison of the metrics in Tables 3 and 4, where cropped images resized to 160 × 160 are used, shows that all the best metrics values are obtained with dataset A. The differences between the obtained global accuracies are statistically significant. As stated by Yap et al. [10], we believe that high quality images (dataset B) may include other structures such as ribs, pectoral muscle or air in the lungs, making the lesion segmentation more difficult.

In Tables 4, 5, and 6 we also compared three variations of database B: one with cropped images resized to 160 × 160, another with original images resized to 160 × 160 pixels and another with original images resized to 320 × 320 pixels. The comparison showed that some metrics were higher in some than others. However, the global accuracy obtained with original images resized to 160 × 160 pixels is better than those obtained with the other two image sets, and the differences are statistically significant.

Comparing this study with other studies using conventional machine learning techniques we noticed that: Infantosi et al. [16], using a subset of dataset A, composed of 200 images, doing the lesion segmentation with morphologic operators and a gaussian function constraint, observed that 91% of the images presented an IoU better than 0.5. Gomez et al. [17] using a subset of dataset A, composed of 50 images, doing the lesion segmentation using marker-controlled watershed transformation, obtained an IoU value of 0.86 ± 0.05. In this study, with CNN3 and dataset A, we obtained a mean IoU of 0.876 and a weighted IoU of 0.920; Jiang et al. [7], as previously cited, obtained an Accuracy of 87.5% using a different dataset. In this study, with CNN3 and dataset A, we obtained an accuracy of 0.950 ± 0.006 and a global accuracy of 0.956 ± 0.011. Torbati et al. [8], as previously cited, obtained an IoU of 86.95% using a dataset with 30 images.

## Conclusion

In this study we evaluated the performance of CNN architectures in the task of breast lesion segmentation in US images. Our main concern was to assess the ability of CNNs to detect contour irregularities in breast lesions in US images.

A qualitative analysis showed that DAG architectures better represent the irregularities present in the gold-standard contours traced by a specialist. The best results were obtained with the more complex DAG architecture.

As future work, we propose evaluating DAG architectures in a large database, which would enable a better network generalization.

## Data Availability

The data that support this study will be provided upon request to the authors, only for academic research purposes and with the commitment to cite this work.

## Abbreviations

BatchNorm: Batch Normalization
BF: Boundary F1
BUS: Breast Ultrasound Images
CAD: Computer-Aided Diagnosis
CNN: Convolutional Neural Network
Conv: Convolutional
CPU: Central Processor Unit
DAG: Direct Acyclic Graph
Deconv: Deconvolutional
DM: Digital mammography
FCN: Fully Convolutional Network
FN: False Negatives
FP: False Positives
GB: Giga Bytes
IoU: Intersection over Union
MaxPooling: Maximum Pooling
RAM: Random Access Memory
ReLU: Rectifier Linear Unit
SGDM: Stochastic Gradient Descent with Momentum
TN: True Negatives
TP: True Positives
US: Ultrasound
